# Supercritical Extraction of Lycopene from Tomato Industrial Wastes with Ethane

**DOI:** 10.3390/molecules17078397

**Published:** 2012-07-11

**Authors:** Beatriz P. Nobre, Luisa Gouveia, Patricia G. S. Matos, Ana F. Cristino, António F. Palavra, Rui L. Mendes

**Affiliations:** 1 Unidade de Bioenergia, LNEG, Estrada do Paço do Lumiar, 1649-038 Lisboa, Portugal; 2 Centro Química Estrutural, DEQB, IST, Av. Rovisco Pais, 1, 1049-001 Lisboa, Portugal; 3 Centro de Ciências Moleculares e Materiais, DQB, FCUL, Campo Grande, 1749-016 Lisboa, Portugal

**Keywords:** carotenoids, ethane, lycopene extraction, supercritical fluid

## Abstract

Supercritical fluid extraction of all*-E*-lycopene from tomato industrial wastes (mixture of skins and seeds) was carried out in a semi-continuous flow apparatus using ethane as supercritical solvent. The effect of pressure, temperature, feed particle size, solvent superficial velocity and matrix initial composition was evaluated. Moreover, the yield of the extraction was compared with that obtained with other supercritical solvents (supercritical CO_2_ and a near critical mixture of ethane and propane). The recovery of all*-E*-lycopene increased with pressure, decreased with the increase of the particle size in the initial stages of the extraction and was not practically affected by the solvent superficial velocity. The effect of the temperature was more complex. When the temperature increased from 40 to 60 °C the recovery of all-*E*-lycopene increased from 80 to 90%. However, for a further increase to 80 °C, the recovery remained almost the same, indicating that some *E*-*Z* isomerization could have occurred, as well as some degradation of lycopene. The recovery of all-*E*-lycopene was almost the same for feed samples with different all-*E*-lycopene content. Furthermore, when a batch with a higher all-*E*-lycopene content was used, supercritical ethane and a near critical mixture of ethane and propane showed to be better solvents than supercritical CO_2_ leading to a faster extraction with a higher recovery of the carotenoid.

## 1. Introduction

Carotenoids include more than 750 compounds, representing one of the major natural coloring groups, that are responsible for the yellow, orange and red colors of many fruits, vegetables, flowers, fungi, algae and some animals. Lycopene is a lipid soluble carotenoid (C_40_H_56_) which presents *Z*-(*cis*) and *E*-(*trans*) geometrical isomers due to the presence of the methyl groups bonded to a polyenic chain. It is responsible for the red color of tomato and its based products, being the major carotenoid present in this fruit. Other available natural sources of this compound include watermelon, apricot, papaya, guava, pink grape-fruit, pumpkins and rosehip fruit [1,2,3,4,5]. On the other hand, some microorganisms have been reported in the last years as a promising source of this carotenoid [6,7].

Lycopene’s major commercial use is as a colouring agent in the feed, food, nutraceutical and pharmaceutical industries [8], although its biological properties, as anti-oxidant and anti-carcinogenic agent, have been gaining increased attention in the last decade [9]. Due to this fact, lycopene consumption is strongly recommended for reducing the risk of atherosclerosis, coronary heart diseases and some types of cancer [10]. 

Commercially available lycopene is mainly obtained by extraction and purification of tomatoes using hexane and ethyl acetate as solvents or by chemical synthesis [11], although some lycopene-producing microorganisms are also being used on a smaller scale [6,7]. Lycopene content in tomatoes can vary greatly, depending on environmental factors, agricultural techniques and tomato types [12,13], with values ranging between 10 and 200 mg/kg (wet basis). Most of the compound is located in the insoluble fraction with the skin containing five times more lycopene than the pulp [10,14,15]. On the other hand, the lycopene bioavailability increases with cooking (heating) and smashing of the vegetable matrices [16].

Tomato industries produce large amounts of solid wastes, up to 3–4% of the processed fruit [17], consisting mainly of skins and seeds. As a consequence, most of the original lycopene in tomatoes is wasted. On the other hand, these solid residues are frequently used, without further treatment, as animal feed. Therefore, the extraction of this valuable compound could be a good alternative to the valorization of this by-product [18,19].

Supercritical fluid extraction of compounds with interest in the food and nutraceutical industries has received increased attention in the past three decades. It presents important advantages over the conventional organic extraction, such as reducing the use of toxic solvents, the possibility to work at moderate temperatures avoiding thermal degradation of the carotenoids, obtaining a solvent free extract, higher selectivity and in some cases higher yields. Carbon dioxide is the most used supercritical solvent due to its well described properties [20], but other solvents like ethane or propane have also being successfully applied [21,22]. The use of ethane instead of CO_2_ as supercritical solvent for the food industry may offer several advantages, since ethane, although being more expensive than CO_2_, has a low critical temperature, near that of supercritical CO_2_ and a low critical pressure (305.4 K and 48.2 atm [23]) which will allow reducing the energy costs associated to the process [21]. On the other hand, ethane is an apolar solvent, with a higher polarizability than CO_2_ [20], which makes it presumably a better solvent for the type of carotenoids found in tomato.

Supercritical fluid extraction of lycopene from tomato industrial wastes has been recently proposed as an alternative technology [24,25,26,27,28,29,30,31,32]. Baysal *et al*. [25] studied the extraction of lycopene and β-carotene from the by-products of the tomato industry, using supercritical CO_2_. These authors evaluated the effect of pressure, temperature, time of extraction, solvent flow-rate and addition of co-solvent (ethanol).The maximum recovery of lycopene, 54%, was obtained at 55 °C and 300 bar with the addition of 5% (vol.) ethanol as co-solvent. Rozzy *et al*. [24] also extracted lycopene from tomato residues with supercritical CO_2_ and obtained as best extraction conditions 56 °C and 344.7 bar, obtaining 61% of the total lycopene. Sábio *et al*. [28] obtained a recovery of lycopene of 80%, from the skins and seeds of tomatoes, using supercritical CO_2_ at 60 °C and 300 bar. Ollanket *et al.* [31] achieved 100% recovery of lycopene with supercritical CO_2_ at 110 °C and 400 bar. Favati *et al*. [26] obtained a recovery of 64% of lycopene from industrial tomato wastes using supercritical CO_2_ modified with sunflower oil, at 80 °C and 500 bar. Furthermore, Vasapollo *et al.* [27] extracted a maximum of 60% of lycopene from tomato with CO_2_, at 450 bar and 66 °C, using hazelnut oil as co-solvent. The published data show that supercritical fluid extraction is suitable for the extraction of lycopene from tomato industrial wastes. Nevertheless, to the best of our knowledge, none of the published studies deals with the use of ethane as supercritical solvent. 

The objective of this work was to study the effect of several parameters (pressure, temperature, solvent superficial velocity, particle size and matrix initial composition) on the extraction of all-*E*-lycopene from tomato industrial wastes using supercritical ethane. The comparison of the recovery of all-*E*-lycopene using other solvents (supercritical CO_2_ [32] and a near critical mixture of ethane and propane) was also carried out.

## 2. Results and Discussion

### 2.1. Effect of Matrix Composition

Two tomato paste waste matrices, M1 and M2, with different all-*E*-lycopene contents (M1—127 μg/g dry matter; M2—213 μg/g dry matter), were submitted to supercritical ethane extraction at temperature of 60 °C, pressure of 300 bar and solvent superficial velocity of 1.44 cm/min. 

**Figure 1:**
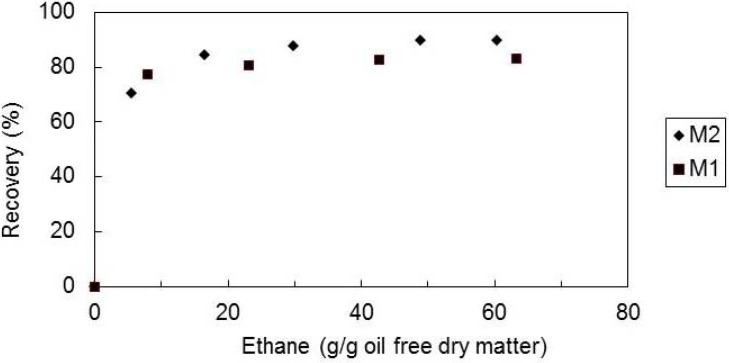
Recovery of all-*E*-lycopene as a function of ethane amount, at 60 °C, 300 bar, matrix particle size of 0.36 mm and solvent superficial velocity of 1.44 cm/min, for the two tomato waste matrices tested (M1 and M2).

Figure 1 shows the recovery (mass of extracted compound by supercritical fluid extraction/mass of extracted compound by Soxhlet × 100) of all-*E*-lycopene from the two matrices, M1 and M2. It can be seen that the recovery of all-*E*-lycopene is almost the same for both matrices. This behavior is different than that obtained with supercritical CO_2_, in which matrices with higher lycopene content showed a slower extraction rate and a lower recovery of the carotenoid [32]. The extraction curves show that in the first period of the extraction (when the process is possibly controlled by the equilibrium between the supercritical ethane and the solutes in the solid matrix) almost all the lycopene is extracted. The higher solubility of lycopene in ethane (when compared with that in CO_2_) will allow a faster extraction. For the matrix with higher lycopene content possibly a higher amount of lycopene is accessible to the solvent and so more carotenoid will be extracted. 

### 2.2. Effect of the Supercritical Solvent

**Figure 2:**
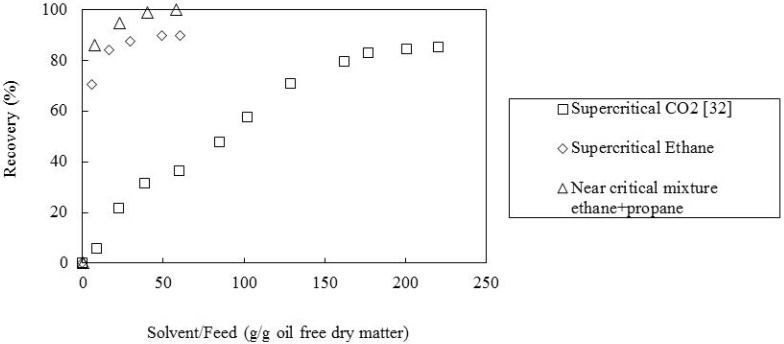
Recovery of all-*E*-lycopene from sample M2 as a function of solvent amount, at 60 °C, 300 bar, matrix particle size of 0.36 mm and solvent superficial velocity of 1.44 cm/min, for the solvents tested.

The sample of tomato paste waste M2 was also submitted to supercritical fluid extraction using supercritical CO_2_ [32] and a near critical mixture of ethane and propane. In Figure 2 the obtained recoveries are shown. It was verified that the mixture of ethane/propane led to the higher recovery (about 100%) of all-*E*-lycopene, followed by ethane (90%) and CO_2_ [32] (86%). Also the extraction was faster when ethane and the mixture of ethane and propane were used. This behavior could be explained in terms of the solubility of the compound in the supercritical solvent which will be higher in ethane and in the mixture of ethane + propane than in CO_2_. Suogi *et al.* [29] carried out supercritical fluid extraction of lycopene from tomato industrial wastes using CO_2_ and propane as solvents and verified that the extraction using propane required a lower amount of solvent than that using CO_2_. Furthermore, it was possible to work at lower pressures with the former solvent. On the other hand, Nobre *et al.* [33,34] found also that supercritical ethane and near critical mixtures of ethane and propane were better solvents for β-carotene (a structural isomer of lycopene) than CO_2_. Moreover, the solubility of this carotenoid increased with the amount of propane in the mixture of solvents. 

### 2.3. Effect of the Pressure and Temperature

**Figure 3:**
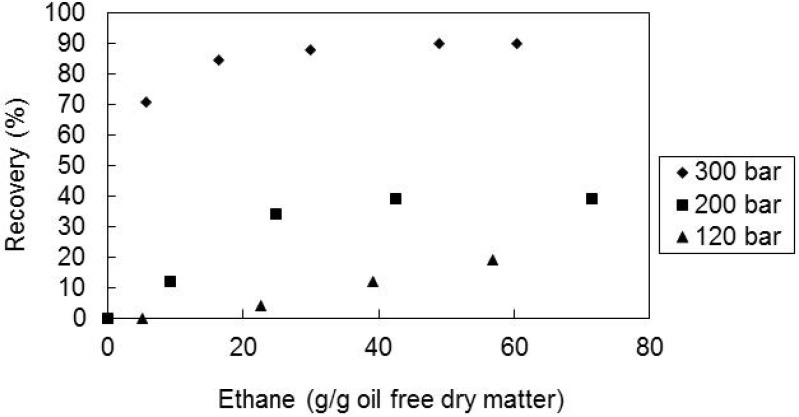
Recovery of all-*E*-lycopene from sample M2 as a function of solvent amount, at 60 °C, solvent superficial velocity of 1.44 cm/min and matrix particle size of 0.36 mm for the several pressures tested.

The effect of pressure on the recovery of the extraction was also studied. In Figure 3 is represented the recovery of all-*E*-lycopene from matrix M2, at temperature of 60 °C, superficial velocity of 1.44 cm/min and pressures of 120, 200 and 300 bar. It can be seen that the recovery increases with pressure and rises drastically when the pressure increases from 200 to 300 bar, which is expected due to the increase in the solvent density with pressure. Also, the extraction is much faster at the highest pressure. This could possibly be due to the fact that the extraction of carotenoids occurred only after most of the lipids, mainly triglycerides, were extracted at 200 bar.

**Figure 4:**
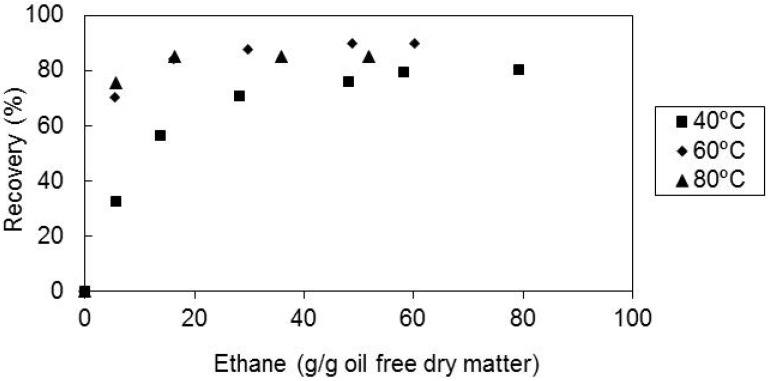
Recovery of all-*E*-lycopene from sample M2 as a function of solvent amount, at 300 bar, solvent superficial velocity of 1.44 cm/min and feed particle size of 0.36 mm, for the several temperatures tested.

The effect of temperature in the recovery of all-*E*-lycopene was also studied and the results, expressed as recovery of all-*E*-lycopene versus temperature are represented in Figure 4. It is shown that the recovery of the carotenoid increased slightly when the temperature increased from 40 to 60 °C and remained almost the same with a further rise of the temperature to 80 °C. That increase is possibly due to some all-*E- > Z* isomerization occurred at the higher temperature used, since the supercritical extracts showed a higher amount of the Z isomer at this temperature.

### 2.4. Effect of the Solvent Superficial Velocity and Particle Size

To study the effect of the ethane superficial velocity through the extractor, supercritical fluid extraction experiments were carried out at the temperature of 60 °C, pressure of 300 bar, mean particle size of 0.36 mm. Three different solvent superficial velocities were used: 0.63, 1.44 and 2.58 cm/min. 

**Figure 5:**
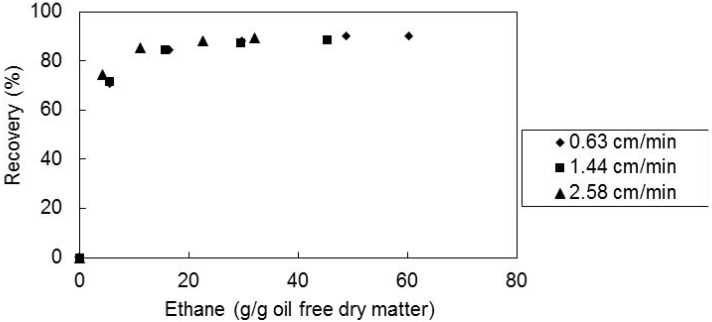
Recovery of all-*E*-lycopene from sample M2 as a function of solvent amount, at 60 °C, 300 bar and matrix particle size of 0.36 mm for the several solvent superficial velocities.

In Figure 5 are shown the obtained recoveries of *E*-lycopene as a function of the solvent amount. It can be observed that the final recoveries are independent of the three supercritical ethane flowrates, although, in the initial period of extraction the recovery increases slightly when the flow rate decreases.

Moreover, the effect of the particle size on the supercritical extraction behaviour was also studied. Three different mean particle size samples of the tomato paste waste (0.15, 0.36 and 0.56 mm) were used in supercritical fluid extraction with ethane at 60 °C and 300 bar. 

**Figure 6:**
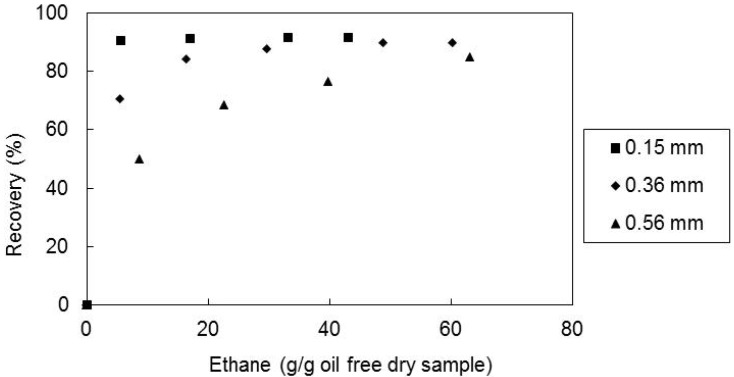
Recovery of all-*E*-lycopene from sample M2 as a function of solvent amount, at 60 °C, 300 bar and solvent superficial velocity of 1.44 cm/min, for the several matrix particle sizes tested.

In Figure 6 the recoveries of all-*E*-lycopene are shown as a function of the solvent amount. It was found that the final recovery of all-*E*-lycopene is practically independent of the particle sizes used in this work. The reduction of size increased the particle surface area and also can cause ruptures of the cell walls becoming the all-*E*-lycopene more accessible to the supercritical solvent in the initial stages of extraction.

## 3. Experimental

### 3.1. Materials

Ethane (99.998% purity) was purchased from Air Liquide (Lisbon, Portugal). A solvent mixture of ethane and propane (99.995% purity) with 50%/50% (mole) was obtained from Praxair (Lisbon, Portugal). Tomato industrial wastes (a mixture of skins and seeds) were offered by a local tomato processing company: Fomento da Industria do Tomate SA—FIT. Lycopene standard (90–95% purity) was purchased from Sigma-Aldrich Chemie Gmbh (Steinheim, Germany). Acetone (p.a.), methanol (HPLC grade), acetonitrile (HPLC grade), and *n*-hexane (p.a) were obtained from Merck (Damstadt, Germany). 

### 3.2. Sample Preparation

Two samples of tomato industrial waste were used, M1 and M2. The samples were collected in different occasions and from different types of tomato. Both samples were dried in an oven at the temperature of 40 °C up to a moisture content of 4.6%. The dried tomato pastes were then packed under nitrogen and stored at −20 °C. The product was ground prior to the supercritical fluid extraction measurements using a cutting mill and samples with mean particle size of 0.15, 0.36 and 0.56 mm were used for batch M2 and with particle size of 0.36 mm for batch M1. The amount of all-*E*-lycopene of the tomato waste was determined by Soxhlet extraction of 1 g of ground tomato waste using as solvent a mixture of acetone:hexane (1:1) for 6 hours and quantified by HPLC. The values obtained were: 127 μg/g _dry matter_ for matrix M1 and 213 μg/g _dry matter_ for matrix M2.

### 3.3. Experimental Procedure

The supercritical measurements were carried out with a flow-type apparatus for the extraction of carotenoids from microalgae, whose details were described before [35]. In this apparatus, the metering pump compresses the liquid solvent to the desired pressure, which was controlled by a back-pressure regulator. In order to guarantee that the fluid reaches the extraction vessel at the desired temperature, the fluid passed through a heat exchanger immersed in a temperature-controlled water bath. After passing the extraction vessel the fluid was expanded to atmospheric pressure through a three-way valve, and the extract precipitates in the glass wool placed inside a glass U-tube, which was immersed in an ice and salt (NaCl) bath at the temperature of −21 °C. The total time of extraction varied between 3 to 8 h Gas flowrate was monitored by a rotameter and the total volume of gas was measured with a wet test meter. The 5 mL extraction vessel had an internal diameter of 7.9 mm, being filled with about 1.5 g of tomato industrial wastes, packed between two layers of glass wool.

Fractions of 5 to 10 L of expanded gas were collected along the time. Three solvent superficial velocities were tested: 0.63 cm/min, 1.44 cm/min and 2.58 cm/min (flowrate of 0.16, 0.27 and 0.41 g/min).

**Figure 7:**
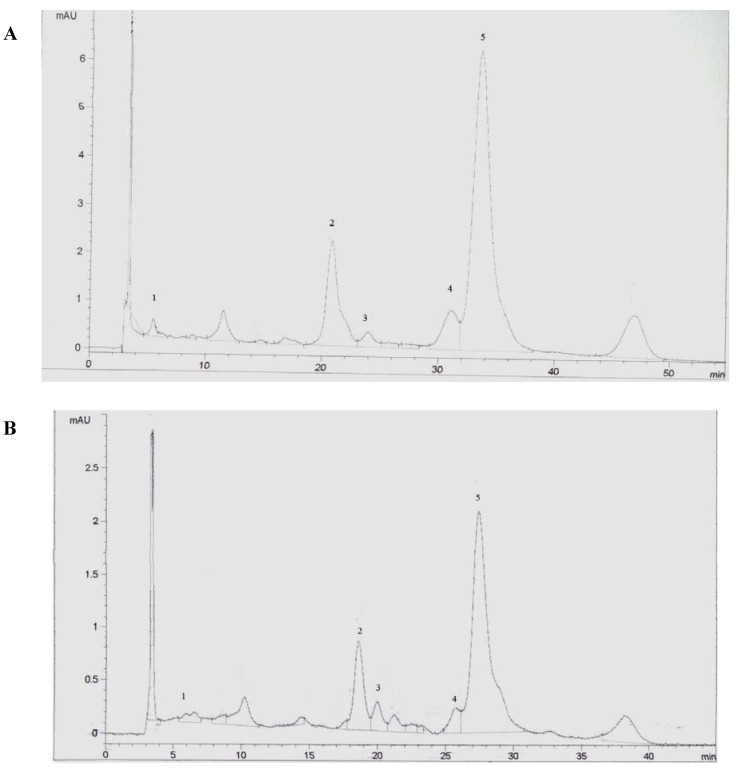
HPLC chromatograms of (**A**) Soxhlet extract; (**B**) supercritical fluid extraction extract (conditions: 60 °C, 300 bar, 0.36 mm and 1.44 cm/min). 1-lutein, 2-all-*E*-beta-carotene, 3-*Z*-beta-carotene, 4-*Z*-lycopene, 5-all-*E*-lycopene.

The extracts were collected by washing the glass wool, the inside of the three-way valve and the expansion tubing with acetone. The collected solutions were analyzed by HPLC in order to quantify the amounts of extracted lycopene. The HPLC system consisted of a Hewlett Packard 1100 series liquid chromatograph, with a UV/VIS detector adjusted to 470 nm. A mobile phase of methanol-acetonitrile (90:10 v/v) was used at 1 mL/min with the reversed phase column, 250 × 4.6 mm, Vydac 201 TP54. All-*E*-lycopene was identified by comparing the retention times of the carotenoid with those of the standard compound, which was also used to obtain calibrations curves, in order to determine the total amounts of all-*E* and *Z*-lycopene. In Figure 7A,B are shown the chromatograms obtained for the Soxhlet and supercritical fluid extracts, respectively.

## 4. Conclusions

Tomato industrial wastes (mixture of skins and seeds) were submitted to supercritical ethane in order to extract all-*E*-lycopene. The recovery of this carotenoid increased with pressure, decreased with the increase of particle size in the initial period of the extraction and was independent of the solvent flow rate. The increase in temperature from 40 to 60 °C leads to an increase in the recovery of all-*E*-lycopene. However, for a higher temperature, 80 °C, the recovery remained almost the same, indicating that possibly some all-*E*-*Z* isomerization (and also some degradation) could have occurred.

On the other hand, the recovery of lycopene was almost the same for samples with different lycopene content. Furthermore, supercritical ethane and the near critical mixture of ethane and propane showed to be better solvents than supercritical CO_2_, leading to a faster extraction and a higher recovery of the carotenoid.
